# Next-Generation Sequencing–Based Cancer Panel Data Conversion Using International Standards to Implement a Clinical Next-Generation Sequencing Research System: Single-Institution Study

**DOI:** 10.2196/14710

**Published:** 2020-04-24

**Authors:** Phillip Park, Soo-Yong Shin, Seog Yun Park, Jeonghee Yun, Chulmin Shin, Jipmin Jung, Kui Son Choi, Hyo Soung Cha

**Affiliations:** 1 Cancer Data Center National Cancer Center Goyang Republic of Korea; 2 Department of Digital Health Samsung Advanced Institute for Health Sciences and Technology Sungkyunkwan University Seoul Republic of Korea; 3 Big Data Research Center Samsung Medical Center Seoul Republic of Korea; 4 Department of Pathology National Cancer Center Goyang Republic of Korea

**Keywords:** data standardization, clinical sequencing data, next-generation sequencing, translational research information system

## Abstract

**Background:**

The analytical capacity and speed of next-generation sequencing (NGS) technology have been improved. Many genetic variants associated with various diseases have been discovered using NGS. Therefore, applying NGS to clinical practice results in precision or personalized medicine. However, as clinical sequencing reports in electronic health records (EHRs) are not structured according to recommended standards, clinical decision support systems have not been fully utilized. In addition, integrating genomic data with clinical data for translational research remains a great challenge.

**Objective:**

To apply international standards to clinical sequencing reports and to develop a clinical research information system to integrate standardized genomic data with clinical data.

**Methods:**

We applied the recently published ISO/TS 20428 standard to 367 clinical sequencing reports generated by panel (91 genes) sequencing in EHRs and implemented a clinical NGS research system by extending the clinical data warehouse to integrate the necessary clinical data for each patient. We also developed a user interface with a clinical research portal and an NGS result viewer.

**Results:**

A single clinical sequencing report with 28 items was restructured into four database tables and 49 entities. As a result, 367 patients’ clinical sequencing data were connected with clinical data in EHRs, such as diagnosis, surgery, and death information. This system can support the development of cohort or case-control datasets as well.

**Conclusions:**

The standardized clinical sequencing data are not only for clinical practice and could be further applied to translational research.

## Introduction

Much research has been conducted to find new biological markers for diagnosis or treatment as next-generation sequencing (NGS) technologies have improved [1]. Recently, as the price and turn-around time of NGS have dramatically reduced, sequencing of patient samples using NGS has been applied in clinical practice [2]. For example, clinical sequencing was mainly applied in cancer patients to determine appropriate treatment by genotyping cancers [3]. Government agencies and private insurance companies in various countries have started to reimburse for clinical sequencing tests. For example, in the United States, if a sequencing laboratory is certified by Clinical Laboratory Improvement Amendments, the sequencing test could be reimbursed [4,5]. Similarly, based on the 100,000 Genomes Project, the National Health Service in the United Kingdom launched a service to provide access to the latest NGS technologies in genomic testing and management [6]. Further, the Korean National Insurance Agency started to reimburse for several panel sequencing tests, including those for cancer and rare diseases, in the beginning of March 2017 [7]. Much additional clinical sequencing has been performed worldwide in clinical practice.

Essentially, clinical sequencing results can be used for diagnosis or to identify appropriate treatment. However, since most of the current clinical sequencing results are not standardized, all clinical sequencing reports are stored in text or pdf format. Therefore, clinical decision support systems cannot utilize the clinical sequencing data through electronic health records (EHRs). In addition, clinical sequencing reports are not interoperable among hospitals owing to the lack of standard adoption. This means that extensive manual manipulation is required for interpretation or use of clinical sequencing reports. Based on the large amount of raw sequencing data and the complicated NGS pipeline from raw data to report generation, clinical sequencing requires well-established standard operating procedures and highly-trained experts to ensure data quality [8]. To resolve this issue, diverse efforts have been made by standard development organizations. ISO/TC 215 focused on clinical genomics by establishing a subcommittee on genome informatics in 2019. It also published two genomics standards [9,10] and developed six genomics standards [11-16]. The HL7 clinical genomics working group also developed diverse clinical genomics standards [17-23].

Given the research problem mentioned above, this study aimed to develop a system to standardize clinical sequencing data and to provide services suitable for researchers to utilize the data. In this study, we extended a clinical NGS research system (CNRS) in a clinical research data warehouse (CRDW) that structures and standardizes clinical sequencing results by mapping standard terminology from current unstructured text reports.

## Methods

### System Architecture and Data Flow

**Figure 1:**
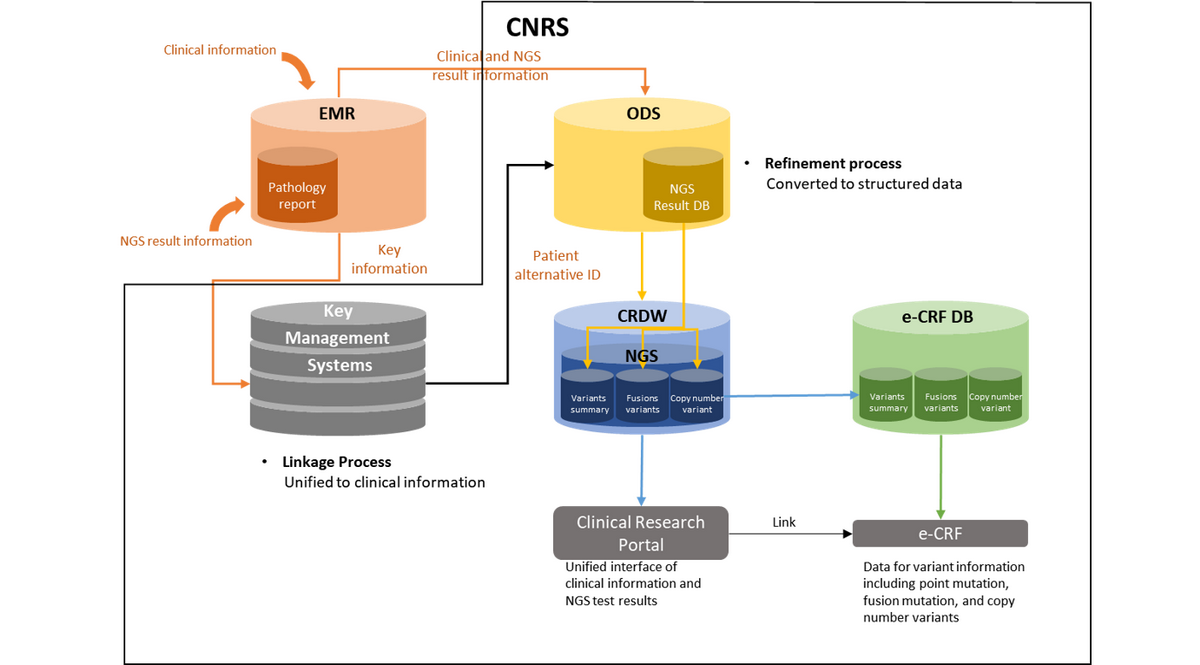
Data flow of the clinical next-generation sequencing (NGS) research system. This system was built for the unified management of the clinical information of each patient and clinical NGS test results. CNRS: clinical next-generation sequencing research system; CRDW: clinical research data warehouse; DB: database; e-CRF: electronic-case report form; EMR: electronic medical record; ODS: operational data store.

Figure 1 depicts the data flow of the CNRS. The clinical sequencing report is stored as a part of the pathology report in EHRs. The stored clinical sequencing reports are transferred to the operational data store (ODS). In ODS, the data are structured and standardized, and the sequencing information is saved in the CRDW. Through a key management server, the clinical data in the CRDW and the NGS result data in pathology reports are mapped to patient alternative numbers and not patient numbers. In addition, NGS result data in the CRDW are extracted and stored in the NGS viewer database (DB). Researchers can access the necessary deidentified specific genetic variation cohort in the clinical research portal and inspect NGS result data in the NGS result viewer.

The detailed information of each component is presented in subsequent sections.

### Data Collection of Clinical Sequencing Results

The Health Insurance Review & Assessment Service in Korea reimburses laboratory-developed panel sequencing tests that include about 100 genes with 14 mandatory genes. As an example, Table 1 shows the panel genes that are used in the National Cancer Center (NCC), Korea.

**Figure 2:**
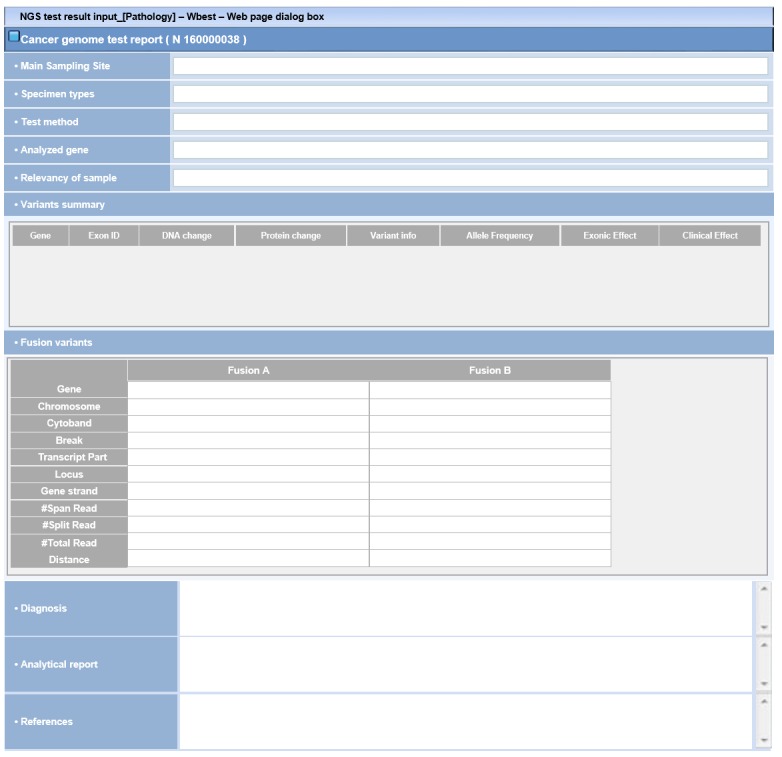
Input template of the clinical sequencing report of the National Cancer Center. All boxes are text boxes for free text entry.

The current clinical sequencing report of the NCC is illustrated in Figure 2. Currently, clinical sequencing reports are stored in a single table with 28 attributes in the EHR DB. This table is copied to the ODS on a weekly basis.

**Table 1:** List of panel genes (n=91) used in the National Cancer Center, Korea.

Category	Genes
Mandatory genes (n=14)	*ALK*, *BRAF*, *BRCA1*, *BRCA2*, *EGFR*, *HER2*, *IDH1*, *IDH2*, *KIT*, *KRAS*, *MYC*, *MYCN*, *NRAS*, and *PDGFRA*
Additional genes (n=74)	*ABL1*, *AKT1*, *AKT3*, *APC*, *AR*, *ATM*, *AXL*, *CCND1*, *CDH1*, *CDK4*, *CDK6*, *CDKN2A*, *CEBPA*, *CSF1R*, *CTNNB1*, *DDR2*, *ERBB2*, *ERBB3*, *ERBB4*, *ERG*, *ESR1*, *ETV1*, *ETV4*, *ETV5*, *EZH2*, *FANCA*, *FANCC*, *FANCF*, *FANCG*, *FBXW7*, *FGFR1*, *FGFR2*, *FGFR3*, *FGFR4*, *FLT3*, *FOXL2*, *GNA11*, *GNAQ*, *GNAS*, *HNF1A*, *JAK1*, *JAK2*, *JAK3*, *KDR*, *MAP2K1*, *MAP2K2*, *MAP2K4*, *MET*, *MLH1*, *MTOR*, *NOTCH1*, *NPM1*, *NTRK1*, *NTRK2*, *NTRK3*, *PIK3CA*, *PIKR1*, *PPARG*, *PTEN*, *PTPN11*, *RAF1*, *RB1*, *RET*, *ROS1*, *RUNX1*, *SMAD4*, *SMARCB1*, *SMO*, *SRC*, *STK11*, *TP53*, *VHL*, *WT1*, and *NRG1*
Additional fusion genes^a^ (n=3)	*ALK*, *ROS1*, and *RET*

^a^Genes in the fusion category are duplicated in the mandatory gene list.

### Structuring/Standardization Process

**Figure 3:**
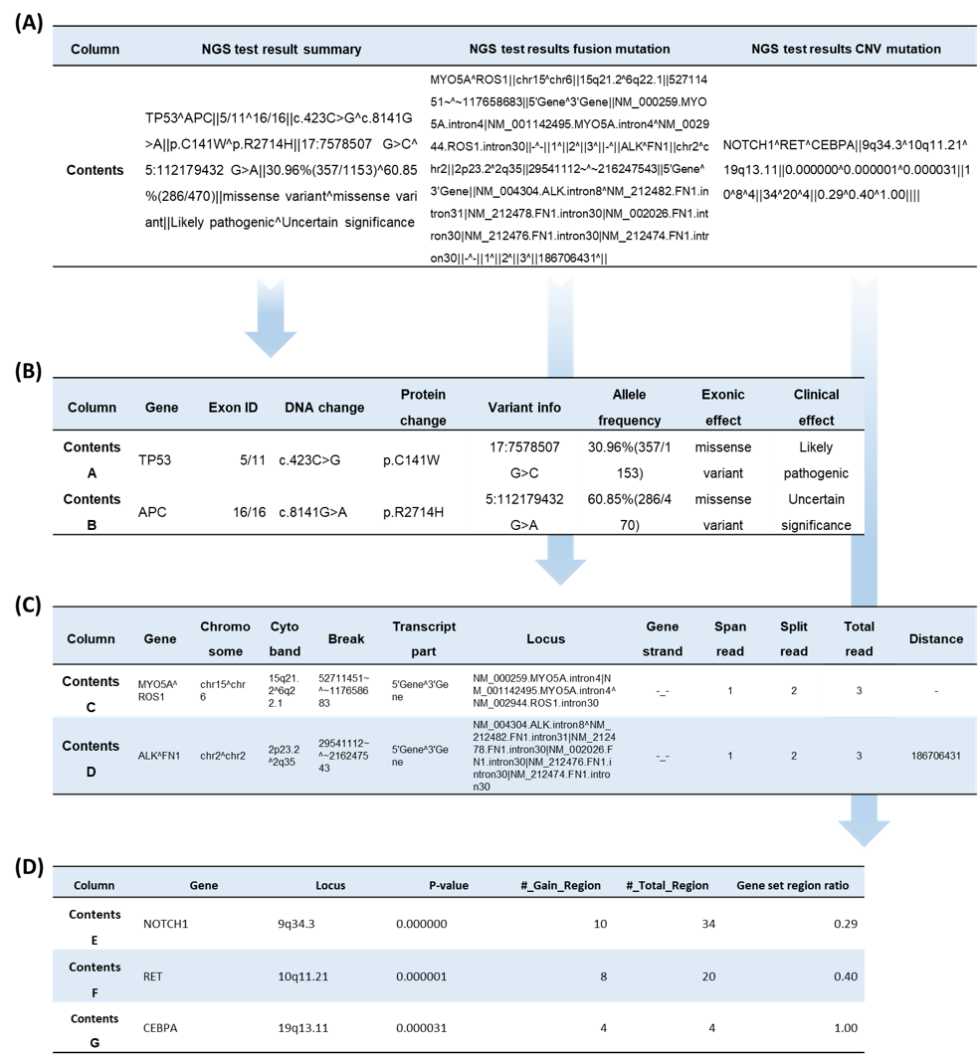
Structure of the next-generation sequencing (NGS) test results. (A) Clinical NGS result summary of the electronic health record database (DB). (B) Variant summary table in the NGS DB. (C) Gene fusion table in the NGS DB. (D) Copy number variation table in the NGS DB.

Figure 3 demonstrates the data structuring process. All data are structured and standardized according to ISO/TS 20428 during the extract transform and load process [10]. The ISO/TS 20428 standard defines the required and optional fields for sequencing reports, along with the metadata for each field. The required fields include the following 10 categories: clinical sequencing orders, information on the subject of care, information on the legally authorized person ordering clinical sequencing, performing laboratory, associated diseases and phenotypes, biomaterial information, genetic variations, classification of variants, recommended treatment, and addendum. The optional fields include the following seven categories: medical history, family history, reference genome version, racial genomic information, genetic variation, detailed sequencing information, and references.

Figure 3 shows that the clinical sequencing data are structured and loaded from the replicated ODS into pathologic and laboratory information, variant summary, fusion variant, and copy number variation (CNV) variant tables. Pathologic and laboratory information has the following nine attributes: identifiers, test order date, quality control results, sample type, report generation date, report generator information, sequencer type, recommended treatment, and references. Variant summary has the following nine attributes: gene name, exon ID, DNA change, protein change, variant information, allele frequency, effects of variants, pathogeny, and clinical relevance. Fusion variant has the following 11 attributes: gene name, chromosome, cytoband, break, transcript part, locus, gene strand, span read, split read, total read, and distance. CNV has the following nine attributes: gene name, locus, *P* value, gain region, total region, region ratio, gene count, region count, and significant region count.

### Combination With Clinical Data

**Figure 4:**
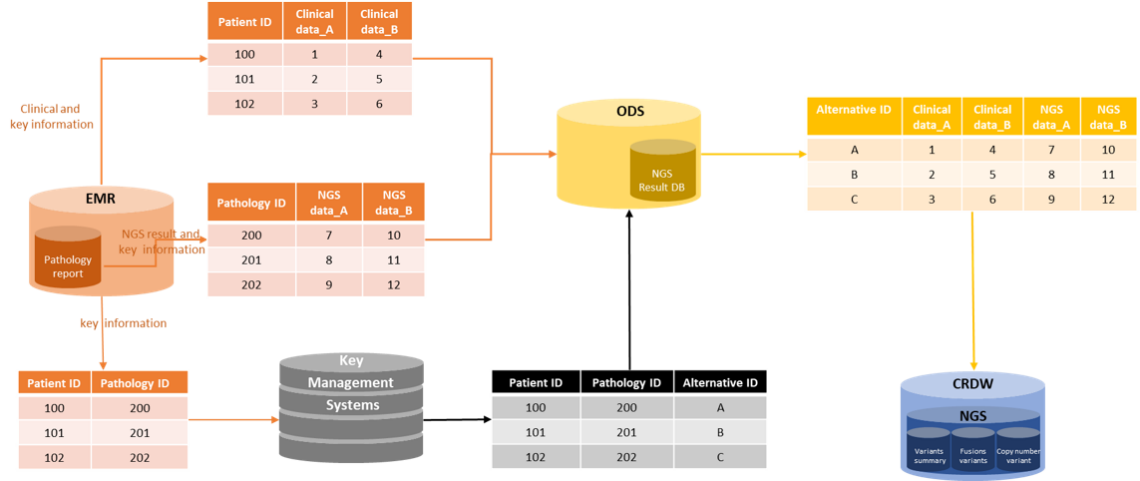
Overview of the combination with clinical data. CRDW: clinical research data warehouse; DB: database; EMR: electronic medical record; NGS: next-generation sequencing; ODS: operational data store.

Figure 4 shows that the NCC has deidentified the clinical data warehouse as well. The ODS receives clinical data from the table with the primary key as the patient ID and NGS result data from the table with the primary key as pathology ID from EHRs. The key management system receives the pathology ID and patient ID from EHRs and generates an alternative ID. It then sends the ID to the ODS. The ODS deletes the patient ID and pathology ID and sends the data to the CRDW with the alternative ID. By using an alternative ID, which is a pseudonym for the patient ID, the necessary deidentified clinical data can be combined with the sequencing result data.

### User Services

Users can access the necessary information using a clinical research portal and NGS result viewer. The clinical research portal is a user interface to query or extract clinical and genomic data by changing search options. The NGS result viewer provides functionality to create a structured or standardized clinical sequencing report by converting an original unstructured pathology report using ISO/TS 20428.

This study was approved by the institutional review board (IRB) of the NCC in Korea (NCC2019-0535).

## Results

**Figure 5:**
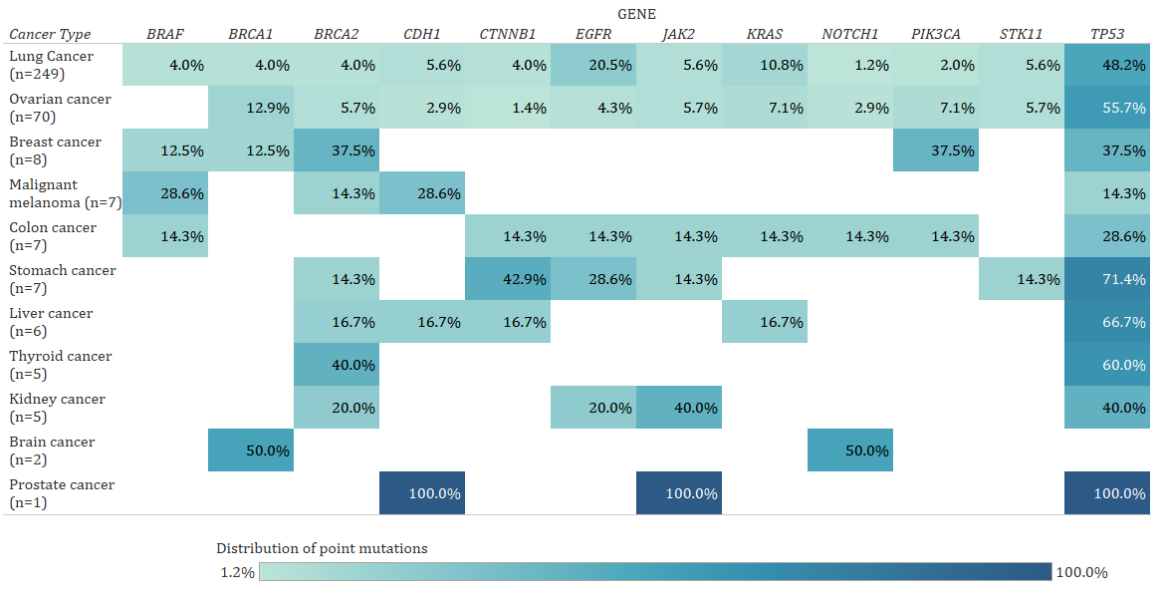
Distribution of point mutations by cancer type in 367 patients. The top 12 genes by frequency are displayed.

From April 2017 to February 2019, the CNRS included 367 clinical sequencing results, which consisted of 249 lung cancer cases, 70 ovarian cancer cases, eight breast cancer cases, seven malignant melanoma cases, seven colon cancer cases, seven stomach cancer cases, six liver cancer cases, five thyroid cancer cases, five kidney cancer cases, two brain cancer cases, and one prostate cancer case. In detail, 51 variants were found and stored among a total of 88 genes. Figure 5 shows the distribution of point mutations by cancer type. Across all cancer types, *TP53* (167/367, 45.5%), *EGRF* (56/367, 15.3%), *KRAS* (34/337, 9.3%), and *BRAC1* (21/337, 5.7%) mutations were common. According to each cancer type, *TP53* (120/249, 48.2%), *EGFR* (51/249, 20.5%), and *KRAS* (27/249, 10.8%) mutations were common in lung cancer; *TP53* (39/70, 55.7%), *BRCA1* (9/70, 12.9%), and *PIK3CA* (5/70, 7.1%) mutations were common in ovarian cancer; and *TP53* (3/8, 37.5%), *BRCA2* (3/8, 37.5%), and *PIK3CA* (3/8, 37.5%) mutations were common in breast cancer. The rates of pathogenic, likely pathogenic, and uncertain significance variants were 55.1%, 34.7%, and 42.9%, respectively.

**Figure 6:**
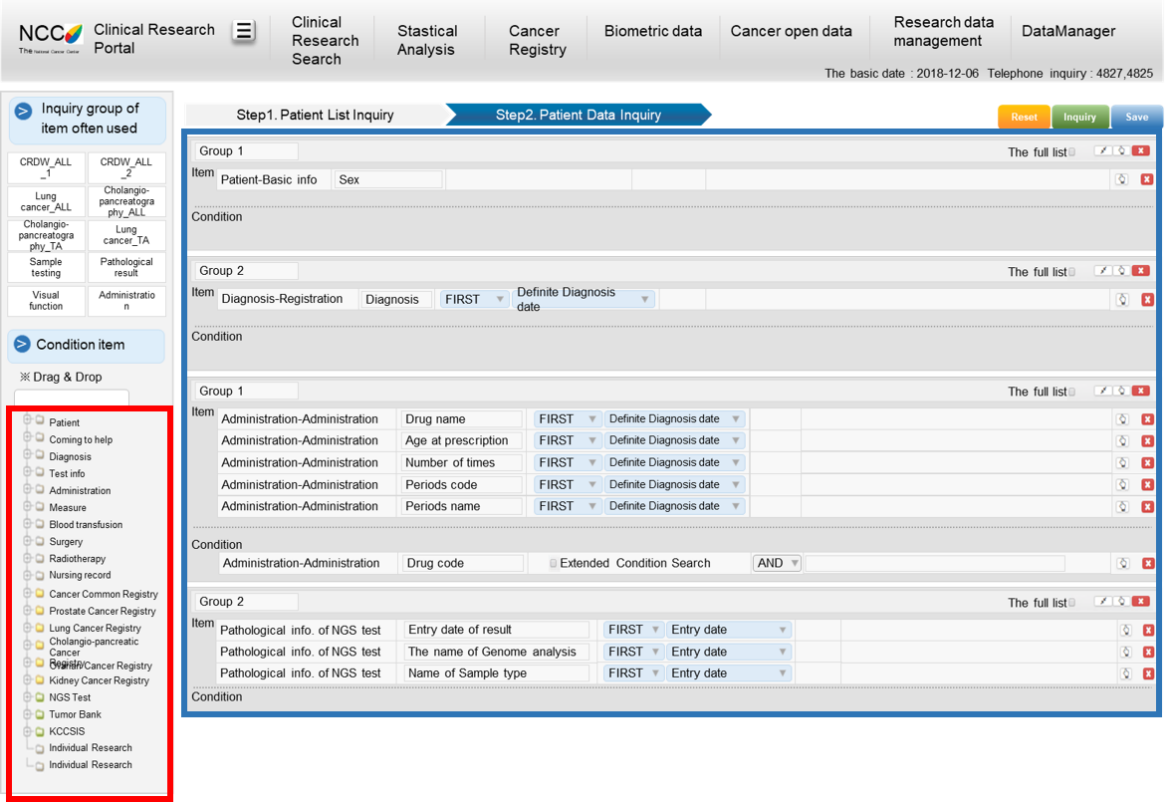
Interface of the clinical research search portal. The main page of the clinical research search portal comprises two domains. The red rectangle indicates searchable items. The blue rectangle indicates the area where the researcher can select items through drag and drop.

**Figure 7:**
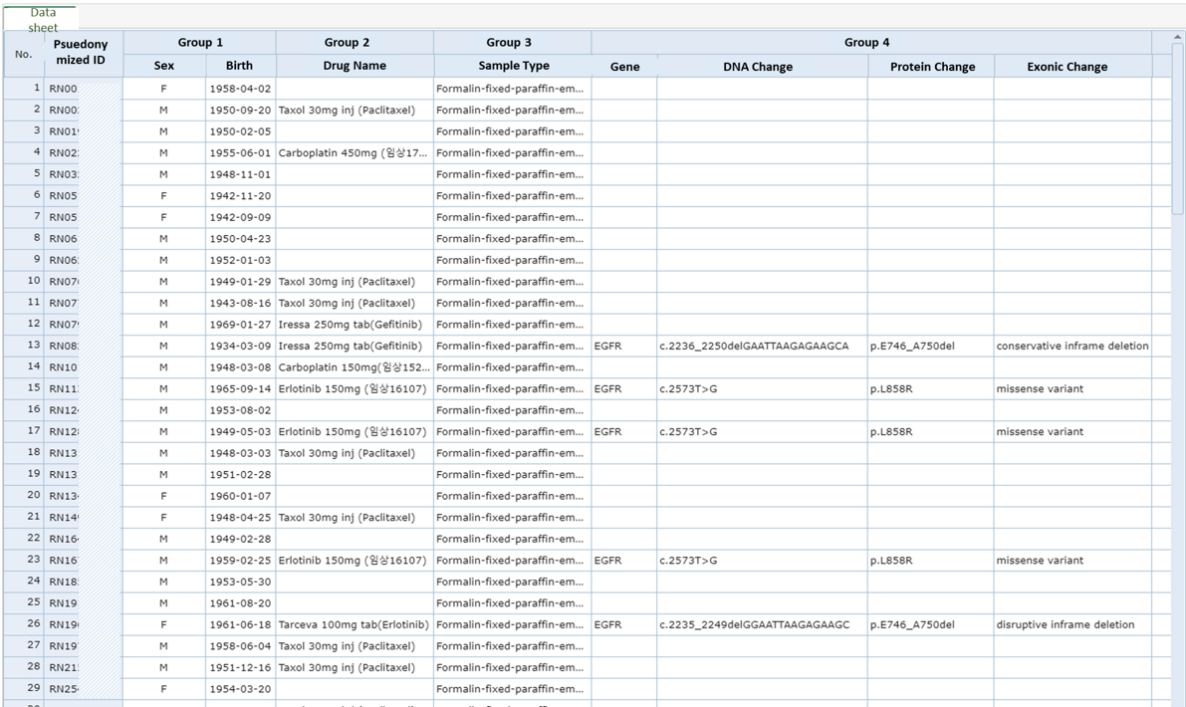
Example of next-generation sequencing results in the clinical research portal.

Figure 6 presents the user interface of the clinical research portal. As explained in the previous section, the clinical research portal supports an integrated view of the NGS results and the corresponding clinical data in EHRs. Figure 6 shows the category of the CRDW, which contains clinical data, such as diagnosis, laboratory data, medication, surgery, chemotherapy, follow-up data, and patient demographic data. Users can choose the appropriate category and then choose desired detailed variables by clicking on them. The NGS results can be visualized (ie, variant summary, CNV, and fusion genes), as illustrated in Figure 7.

**Figure 8:**
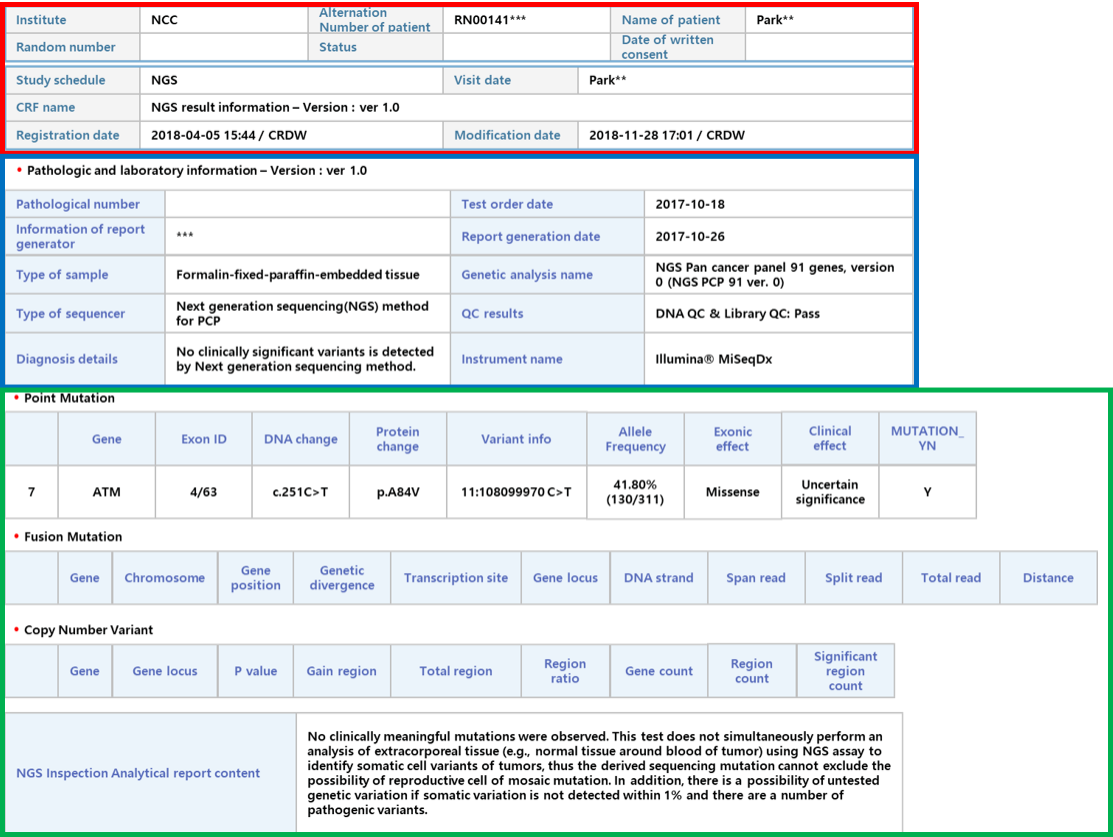
Example of the next-generation sequencing (NGS) result viewer. The main page of the NGS result viewer is composed of three domains. The first box provides basic test information. The second box explains sequencing methods and other related information. The last box shows mutation data with reporting results.

The NGS result viewer can show the detailed clinical sequencing report of each patient in a structured way, whereas the clinical research portal supports the analysis of aggregated sequencing results. As shown in Figure 8, the clinical sequencing report is mainly divided into the following three parts: basic test information, sequencing methods and other related information, and variants with reporting results.

## Discussion

### Principal Findings and Implications

The CNRS converts text-based clinical sequencing reports in EHRs into structured data using international standards. Through the CNRS, the content, as shown in Figure 5, can be easily organized and data can be managed according to international standards. It also provides standardized data through the clinical research search portal; furthermore, using its functions, researchers can set up cohorts with specific mutations and add clinical data columns as needed. Thus, it can be inferred that the CNRS supports researchers in performing translational research by allowing them to easily extract the desired clinical and genomic data from EHRs. For example, non–small cell lung cancer genotyping requires mutation pattern analysis of *KRAS*, *EGFR*, and *BRAF* [1,24]. Similarly, other research requires clinical data such as that on cancer stage, smoking history, and death date for survival analysis [25]. These types of translational studies can be easily performed using the developed CNRS, and this has already been proven by researchers at the NCC.

The CNRS is provided to research projects that have been approved by the IRB. Researchers send data extraction requests to health information managers, and it takes about one to two weeks to review, refine, and provide clinical data from EHRs. The CNRS could retrieve data through the clinical research portal after receiving IRB approval, and it takes about two or three days from review to delivery after a data extraction request is made. In addition, the NCC has built a genomic cohort linking the NGS DB with cancer registries such as those of lung cancer and ovarian cancer.

There are two unique aspects of the CNRS as developed. One is that the CNRS uses an ISO standard (ISO/TS 20428) to support multicenter research. If other hospitals or research institutes use international standards, the data can be easily integrated into the same format. We also achieved the same results as successfully converting the data and manually cleansing the data previously. The other is that the CNRS can help protect patients’ privacy by deidentifying protected health information. To use the patients’ data for research purposes, researchers must obtain written consent from patients or deidentify the identifiable data. In this era of big data, deidentification is usually used for a number of reasons. However, if we deidentify the data, it is difficult to link the separate DBs. To overcome this issue, the CNRS adopted a key management server to pseudonymize the patient ID. This means that the CNRS works as an honest broker [26]. To strengthen the protection level, only authorized developers can access this key management server and users can receive the randomly assigned ID after combing clinical data and sequencing data using the key management system. Therefore, users cannot retrieve real patient IDs in EHRs or pseudonymized IDs in the key management system.

### Limitations

The main contribution of this study is that, for the first time, the ISO/TS 20428 standard was applied to the CRDW to standardize clinical genomic test results. As a result, we also demonstrated that this approach could enable easy search and analysis with clinical data in EHRs. However, it has limitations. We did not verify this system in multiple centers. We hope our approach will help other hospitals or institutions build their own systems.

### Future Work

Our continuing objectives are to extend the categories of clinical and sequencing data in the CNRS and consider the standards proposed by the Global Alliance for Genomics and Health, which has developed diverse practical standard application programming interfaces for international genomic research.

### Conclusion

The CNRS converts the text-based clinical sequencing reports of EHRs into structured data using international standards and provides standardized data. In addition, the CNRS allows researchers to set up cohorts with specific mutations and add clinical data columns as needed. Therefore, it can be inferred that the CNRS enables researchers to conduct translational research by allowing them to easily extract the required clinical and genomic data from EHRs.

## Abbreviations

CNRS: clinical next-generation sequencing research system
CNV: copy number variation
CRDW: clinical research data warehouse
DB: database
EHR: electronic health record
IRB: institutional review board
NCC: National Cancer Center
NGS: next-generation sequencing
ODS: operational data store
